# Targeting shared pathways in tauopathies and age-related macular degeneration: implications for novel therapies

**DOI:** 10.3389/fnagi.2024.1371745

**Published:** 2024-04-03

**Authors:** Michele Rinaldi, Antonio Pezone, Gaia Italia Quadrini, Gianmarco Abbadessa, Maria Paola Laezza, Maria Laura Passaro, Antonio Porcellini, Ciro Costagliola

**Affiliations:** ^1^Department of Neurosciences, Reproductive Sciences and Dentistry, University of Naples Federico II, Naples, Italy; ^2^Department of Biology, University of Naples Federico II, Naples, Italy; ^3^Division of Neurology, Department of Advanced Medical and Surgical Sciences, University of Campania Luigi Vanvitelli, Naples, Italy; ^4^Department of Medicine and Health Sciences “V. Tiberio”, University of Molise, Campobasso, Italy

**Keywords:** tauopathies, age-related macular degeneration, Alzheimer’s disease, drusen, amyloid-β, oxidative stress

## Abstract

The intricate parallels in structure and function between the human retina and the central nervous system designate the retina as a prospective avenue for understanding brain-related processes. This review extensively explores the shared physiopathological mechanisms connecting age-related macular degeneration (AMD) and proteinopathies, with a specific focus on tauopathies. The pivotal involvement of oxidative stress and cellular senescence emerges as key drivers of pathogenesis in both conditions. Uncovering these shared elements not only has the potential to enhance our understanding of intricate neurodegenerative diseases but also sets the stage for pioneering therapeutic approaches in AMD.

## 1 Introduction

Traditionally associated with neurodegenerative disorders like Alzheimer’s disease (AD), the abnormal accumulation of proteins such as tau and amyloid-β in specific brain regions leads to a range of distinct clinical syndromes, each characterized by a unique pattern of neurogeographical involvement. This concept of “neurogeography” is crucial in understanding the specific clinical manifestations of each disorder. AD is the most common neurodegenerative dementia, representing over 50% of all causes of dementia. The pathological hallmarks of AD include the presence of senile plaques, neurofibrillary tangles, neuronal cell loss, reactive gliosis and, in some cases, cerebrovascular amyloid deposits. Senile plaques are generated by deposition and accumulation of the beta-amyloid (Aβ) peptide (Ae and Clark, 2022).

Age-related macular degeneration (AMD) is a progressive decline of retinal pigmented epithelium (RPE) normal function, representing the leading cause of low vision up to central irreversible blindness in industrialized countries (Christoforidis et al., 2011). The pooled global prevalences of early and late-stage disease in adult populations are 8.01% and 0.37%, respectively (Wong et al., 2014). Numerous modifiable and non-modifiable AMD risk factors have been identified, with smoking being the primary modifiable risk factor (Heesterbeek et al., 2020). The earliest visible clinical sign of RPE impairment is the appearance of drusen. Several clinical and pathological characteristics of AD and AMD are similar, indicating overlap in pathology. The phenotypic parallelism between those entities is strongly highlighted by experimental evidence that the Aβ peptide – the main biochemical component of extracellular senile plaques in AD – is also found into ocular drusen in AMD. Moreover, common features include inflammation, hyperphosphorylated tau presence, oxidative stress, and several risk factors such as age, obesity, atherosclerosis, and hypertension. Eventually, in both AMD eyes and AD brains, there are changes in the key protein quality control mechanisms that repair damage caused by oxidative stress. These mechanisms include the autophagic, lysosomal, and proteasomal signal transduction pathways (Kaarniranta et al., 2011). This imbalance in clearance systems likely causes an intracellular buildup in misfolded/damaged proteins, including Aβ and phospho-tau (Ohno-Matsui, 2011), which ends up in the formation of detrimental insoluble aggregates in the brain and in the eye (Bruni et al., 2020). In this review, we will explore the potential parallels between the neurodegenerative processes in diseases like Alzheimer’s and the retinal changes observed in AMD. By examining the latest research and evidence, we aim to provide a comprehensive overview of AMD as a possible proteinopathy, contributing to the broader understanding of these complex diseases.

## 2 The retina: extension of the human brain

The human retina embryologically derives from the diencephalon and shares many features with brain tissue, to the extent that it is reasonably regarded as an extension of the central nervous system. Embryonically, nervous tissue originates from the ectoderm, the outermost primitive germ layer of the embryo, which differentiates into the superficial ectoderm, the neural crest and the neural tube. The neural crest and the neural tube give rise to the neural plate, forming various components of the nervous systems, including the central nervous system, optic nerve, and retina. Around the 18th day of life, an outpouching known as the primary optic vesicle emerges on the neuroectodermal wall of the embryonic forebrain (Darnell and Gilbert, 2016). This structure transforms, evolving into the secondary optic vesicle or optic cup. The optic cup, formed from an infolding of the neuroectodermal wall, consists of two layers, an outer one contributing to the pigmented layer of the retina and an inner one giving rise to the nervous layer, including light-sensing rods and cones. This developmental process concludes by the seventh month, enabling the eye’s sensitivity to light, while the differentiation of the fovea centralis occurs 4 months after birth. Overall, the resemblance in physiological characteristics between the brain and the retina underscores their shared evolutionary origins and interconnected roles in processing sensory information to support various nervous system functions (Mahabadi and Al Khalili, 2023). As neuropathological changes in retinal neurons might mirror brain pathology, a growing hypothesis has suggested its potential role as a “window to the brain” in recent years (Chiquita et al., 2019; Passaro et al., 2023). It engages in intricate processing of visual stimuli before their transmission to the brain. Simultaneously, it processes various facets of the visual environment via parallel pathways directly linked to the occipital cortex, commencing from the optic nerves. Feedback mechanisms between the retina and the brain allow dynamic adjustments in visual processing based on changing environmental conditions. The retina is not merely a passive sensor but actively participates in the neural network responsible for vision. The close integration of the retina with the brain highlights the sophisticated nature of the visual system. In summary, the retina shares structural, developmental, and functional characteristics with the central nervous system, establishing it as an extension of the brain (De Moraes, 2013).

Growing evidence has shown that brain and retinal pathologies share common physio-pathological pathways and clinical features (Trick et al., 1989; Parisi et al., 2001). Several reports have demonstrated the existence of functional changes in the retina of AD patients (Katz et al., 1989; Trick et al., 1989; Parisi et al., 2001; Krasodomska et al., 2010). Those studies, mainly based on pattern electroretinogram (PERG) recordings, reported a decrease in the amplitude and a delay in the latency of the RGCs response, without changes in the visual acuity, trying to establish possible correlations between brain and retina (Trick et al., 1989). Chiquita et al. (2019) reported a decrease of the PERG amplitude in AD patients that is consistent with a delay in the latency of visual evoked potentials (PEV), concluding that the retina could serve as a gateway to further investigate brain pathologies. These conclusions align with the systematic metanalysis conducted by Ge et al. (2021), in which they even state that structural, vascular, and electrophysiological retinal biomarkers hold great potential for the diagnosis, prognosis, and risk assessment of AD and mild cognitive impairment (MCI). They indeed concluded that as thinner retina and choroid, reduced complexity of vessels, and reduced blood flow were found in the MCI stage, retinal biomarkers have great potential utility for early neurodegenerative disease detection (Ge et al., 2021). All these findings highlight the close physiopathological connection between these two systems, clearly defining the importance of examining retinal anatomy and physiopathology to better understand neurodegenerative pathologies and vice versa. They, therefore, plainly indicate the need for further investigation to better understand their significance and, thus, the therapeutic implications. Is it possible to evaluate a synergistic pharmacological approach in aging pathology that has a real impact on processes common to both?

## 3 Proteinopathies: tau and beta-amyloid in neurodegenerative diseases

### 3.1 Introduction to proteinopathies

Proteinopathies are a group of neurodegenerative disorders characterized by abnormal protein accumulation in specific brain regions, leading to distinct clinical syndromes (Cummings et al., 2023). This strict relationship between clinical syndrome and regional accumulation gives rise to the concept of “neurogeography,” emphasizing the significance of the location of these protein build-ups in determining the unique clinical presentations of each disorder (Cummings et al., 2023). For example, Parkinson’s disease primarily affects the substantia nigra, while Dementia with Lewy Bodies impacts the substantia nigra, limbic system, and neocortex. Progressive supranuclear palsy (PSP) shows subcortical tau deposits, corticobasal degeneration (CBD) has asymmetric cortical tau deposits, Huntington’s disease affects the caudate nucleus, AD begins in the hippocampus and spreads, amyotrophic lateral sclerosis targets motor neurons in the spinal cord and motor cortex, and frontotemporal dementia (FTD) affects the prefrontal and orbitofrontal cortex, as well as anterior and medial temporal regions (Chiti and Dobson, 2017; Dugger and Dickson, 2017; Jack et al., 2018). The predominant neuropathological localization in each condition results in corresponding clinical manifestations, often with overlapping symptoms.

### 3.2 Understanding tauopathies: tau protein basics and classification

Among proteinopathies, tauopathies are particularly notable. Tauopathies are characterized by aggregation of tau protein in specific brain regions, resulting in a variety of neurodegenerative diseases (Zhang et al., 2022). Under normal conditions, tau protein, encoded by the microtubule-associated protein tau (MAPT) gene, is primarily located in axons, playing a key role in microtubule assembly and stability (Trabzuni et al., 2012). Through alternative splicing of its gene, tau manifests as six distinct isoforms, with exon 10 determining the presence of either three or four microtubule-binding repeat domains (3R or 4R isoforms) (Zhang et al., 2022). Tauopathies are, therefore, classified into three main categories based on the predominant tau isoform in their aggregates: 3R tauopathies (e.g., FTD), 4R tauopathies (e.g., PSP and CBD), and mixed 3R/4R tauopathies such as AD and chronic traumatic encephalopathy. The concept of neurogeography is clearly exemplified in tauopathies, where each variant displays a unique pattern of brain involvement that shapes its clinical presentation. Key examples include PSP and CBD, both with 4R tau isoforms affecting movement and cognition. Other forms, like FTD and Pick’s disease, vary in tau isoform composition and impact areas like the frontal and temporal lobes, influencing behavior and memory. Conditions such as CTE and argyrophilic grain disease further highlight the diversity of tauopathies. AD, a secondary tauopathy, demonstrates how tau protein aggregation in the brain correlates with cognitive and behavioral symptoms, emphasizing the critical role of specific brain region involvement in disease manifestation (Kovacs et al., 2008, 2020; Léger and Banks, 2014; Irwin et al., 2016; Elahi and Miller, 2017; Hansra et al., 2019; Sakurai et al., 2019; Vasileiou et al., 2019; Busche and Hyman, 2020; Catarina Silva and Haggarty, 2020; Cherry et al., 2020; Jabbari et al., 2020; Murley et al., 2020; Valentino et al., 2020; Humphrey et al., 2021; Von Zglinicki et al., 2021; Cummings et al., 2023).

### 3.3 Alzheimer’s disease: a mixed proteinopathy

Alzheimer’s disease is a complex and multifaceted neurodegenerative condition, and its intricate relationship with different tauopathies highlights its unique pathological characteristics. In the context of tauopathies, AD stands out as a mixed 3R/4R tauopathy, which involves both 3R and 4R isoforms of the tau protein (Hansra et al., 2019). These tau protein aggregates, characteristic of tauopathies, play a crucial role in AD progression. Specifically, AD’s neurodegenerative process is driven by the interplay of Aβ peptides and tau proteins, which are central to its pathogenesis (Shakir and Dugger, 2022). In AD, the brain undergoes significant structural changes marked by the formation of extracellular amyloid plaques and intraneuronal neurofibrillary tangles (Shakir and Dugger, 2022). This combination is significant because it dictates the specific pattern of brain involvement and clinical presentation in AD. The presence of these tau isoforms, along with the amyloid plaques formed by Aβ peptides, is directly linked to the behavioral symptoms of AD, including memory loss and cognitive decline. These symptoms arise primarily due to the damage and loss of synapses, which are vital for cognitive functions.

Recent research over the past 12 years has shown that soluble forms of Aβ and tau collaborate in driving the transition of healthy neurons to a diseased state in AD, independent of their aggregation into plaques and tangles (Bloom, 2014; Busche and Hyman, 2020). The toxic characteristics of Aβ are significantly influenced by tau proteins, with neuron death and synaptic dysfunction triggered by these soluble, toxic forms. In the development of AD, Aβ is upstream of tau, inducing its transformation from a normal to a toxic state (Bloom, 2014). However, this relationship is complex and bidirectional, as toxic tau also enhances Aβ toxicity, creating a feedback loop (Bloom, 2014; Busche and Hyman, 2020).

## 4 Age-related macular degeneration

Age-related macular degeneration, a gradually progressive neurodegenerative retinal condition, has swiftly emerged as the primary cause of vision impairment among the elderly in developed countries, affecting the macular region of the retina (Katz et al., 1989; Trick et al., 1989). The AMD etiology is known to be multifactorial, in addition to a strong genetic component, environmental risk factors such as smoking, obesity, arteriosclerosis, hypertension, and hypercholesterolemia may predispose to AMD (Klein et al., 2004; Katta et al., 2009). AMD is categorized into two types: dry and wet forms. Dry AMD, also known as non-exudative AMD, is marked by the presence of drusen, which are lipid-rich extracellular deposits. In advanced stages, non-exudative AMD, referred to as called geographic atrophy (GA), leads to a gradual deterioration of the RPE and subsequent loss of photoreceptors. On the other hand, wet AMD, or exudative AMD, is characterized by choroidal neovascularization (Xu et al., 2018). The features and pattern of amyloid deposition in drusen and sub-RPE deposits have been investigated in subsequent years, alongside efforts to thoroughly define the proteome of these deposits. Dentchev et al. (2003) characterized the distribution of beta-amyloid in drusen from AMD and normal post-mortem human retinas, finding drusen containing Aβ just in AMD patients’ eyes. Moreover, they found that the number of Aβ positive drusen correlated with the stage of the disease and the coexistence of GA (Dentchev et al., 2003). It is noteworthy that drusen share common molecular constituents with AD amyloid plaques, such as Aβ, vitronectin, apolipoprotein E complement components and inflammatory mediators (Isas et al., 2010). Finally, it is well known that aging causes the decrease of hydraulic conductivity in Bruch’s membrane and changes in lipid content in the retina (Miller-Thomas et al., 2016). Parallel to age, chronic oxidative stress can induce RPE senescence. The synthesis of beta-amyloid is most likely both the cause and the result of these occurrences taken together. Thus, the release of proteases and proinflammatory cytokines results in an irreversible positive feedback process (Feng et al., 2023). According to recent research, inflammation and chronic oxidative stress are strongly linked to AMD and AD pathogenesis (Beatty et al., 2000; Kaarniranta et al., 2011). Complement activation serves as a central mechanism in both AMD and AD pathology models. However, while the classical pathway is thought to play a major role in AD, the alternative pathway is predominantly involved in AMD (McGeer et al., 2005).

## 5 Proofs of link

**FIGURE 1:**
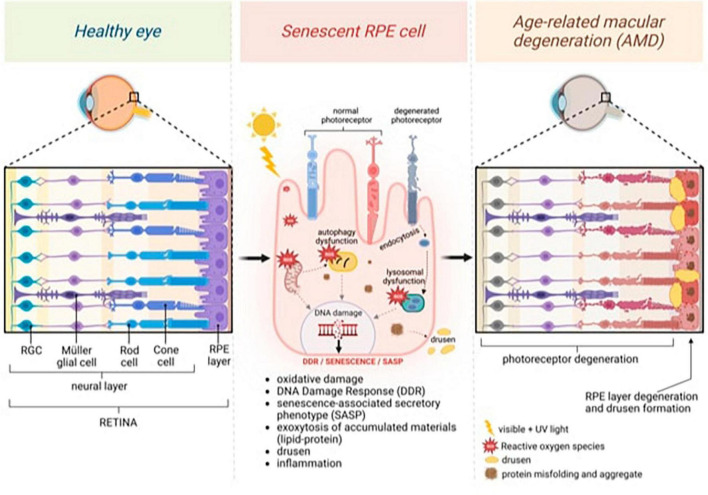
General model of age-related macular degeneration (AMD) featuring changes in the neural and retinal layers. Continuous light exposure and/or deteriorated photoreceptor outer segment fragments (POS) phagocytosis by aged retinal pigment epithelium (RPE) raise oxidative stress in the RPE, damaging nuclear and mitochondrial DNA, and activating DNA damage Response (DDR). Excessive oxidative stress also causes misfolded protein aggregates through impaired lysosomal function and proteasome damage, reducing the protein-repairing process, which, along with malfunctioning autophagy, forms protein aggregates that decrease protein clearance in RPE. Senescence, which is induced by persistent DNA damage and the impaired clearance of toxic accumulations in aged RPE cells, is responsible for inducing senescence-associated secretory phenotype (SASP) and inflammation. Exocytosing protein aggregates causes drusen. Created with BioRender.com.

Tauopathies and retinal degeneration share common physio-pathological pathways, a notion substantiated by robust evidence in the literature (Figure 1; Biscetti et al., 2017; Xu et al., 2018; Wang and Mao, 2021).

### 5.1 B eta-amyloid accumulation

Kam et al. (2010) demonstrated age-related accumulation of Aβ in the mouse retina and human retina. In this article, the accumulation of Aβ (a protein implicated in Alzheimer’s pathogenesis) is identified in the retinas and brains of aged individuals, demonstrating that the pathogenetic process of aging is similar in both retinal and cerebral pathologies. Aβ accumulation primarily occurred among the photoreceptor outer segments and on the interface between the RPE and Bruch’s membrane (Kam et al., 2010; Lynn et al., 2021), suggesting a correlation with the decline in the efficiency of the RPE phagocytotic process (Ning et al., 2008; Gupta et al., 2016). The influence of Aβ accumulation on the outer segments’ photoreceptor loss remains undetermined. Aβ is additionally found in the vascular network of both the inner and outer retina. Analysis of mouse tissue through Western blot revealed that the predominant Aβ types in the retina and RPE-choroidal tissues are the 22e36 kDa and 50e64 kDa oligomers (Kam et al., 2010; Lynn et al., 2017). Later in 2002, Johnson et al. (2002) were pioneers in suggesting that the deposition of Aβ could be a crucial element in local inflammatory events contributing to the atrophy of the RPE and the pathogenesis of AMD. Aβ has indeed been implicated as the primary activator of the complement system in AD. It is associated with a substructural vesicular component within drusen, where it colocalizes with activated complement components, identifying these structures as potential primary sites for complement activation. Thus, the authors highlight the potential involvement of Aβ in contributing to the inflammatory processes associated with AMD development (Johnson et al., 2002; Lynn et al., 2017). In this investigation, the gathered evidence strongly indicates that amyloid vesicles originate from RPE. The RPE cytoplasm displays significant immunoreactivity to amyloid precursor protein (APP), and Aβ immunoreactivity is frequently observed in the cytoplasm of RPE cells surrounding or overlying drusen. Additionally, intracellular structures labeled with anti-Aβ, resembling amyloid vesicles, are identifiable in the RPE cell cytoplasm. Moreover, when labeled with APP and Aβ antibodies, cultured human RPE cells exhibit transcripts for all three APP isoforms and β-secretase. These findings consistently support the proposition that the RPE can produce substantial amounts of APP and generate Aβ through enzymatic processing (Johnson et al., 2002). Additionally, Ding et al. (2008) discovered the presence of Aβ peptide-derived amyloid in sub-RPE deposits and CNV in human eyes affected by AMD.

Later in 2010, Isas et al. (2010) explored the existence of amyloid fibrils in drusen using confocal immunofluorescence microscopy and electron microscopy. The authors employed for their research the OC antibody – derived from immunization with a morphologically consistent population of sonicated Ab42 fibrils – as well as WO1 and WO2 monoclonal antibodies, all known for their specific reactivity to mature amyloid fibrils. OC displayed significant reactivity with AD plaques, and WO antibodies stained sub-RPE deposits, confirming the presence of amyloid structure in drusen vesicles and suggesting the existence of amyloid fibrils in this compartment (Isas et al., 2010). Koronyo-Hamaoui et al. (2011) assessed the presence of Aβ-specific plaques in retinas of AD patients, not only post-mortem but also non-invasively, using curcumin intravenously or orally. Along this line, Chiu et al. reported a series of studies on transgenic mice retina concluding that ocular disease models are of great importance to the investigation of tauopathies pathogenesis (Kurji et al., 2010; Chiu et al., 2012; Chang et al., 2014). All these findings highlight that – despite the well-established association of Aβ with AD – Aβ is detectable in the human retina even in individuals without a history of neurological disease. This demonstrates that the pathogenetic processes of aging are comparable in both retinal and cerebral pathologies.

### 5.2 Hyperphosphorylated tau

Zhao et al. (2013) investigated phosphorylated tau (pTau) expression in AD transgenic mice eyes. The authors found a hyper-expression of pTau in the retina, associated with a significant increase in the production of p35 and p25, and upregulation of calpain, underlining the potential role of hyperphosphorylated tau as a retinal hallmark of AD progression (Zhao et al., 2013). den Haan et al. (2018) assessed the presence of pTau, Aβ, and APP in post-mortem retinas in six AD and six control cases through immunohistochemical staining. Their findings revealed an elevated immunoreactive signal for pTau in both the inner and outer plexiform layers of the retina in AD cases compared to control cases. However, notably, the study did not identify significant Aβ/APP-related differences in the retina between individuals with AD and control subjects (den Haan et al., 2018). Chang et al. (2014) and Lad et al. (2018) obtained comparable results in their exploration of AD and Tauopathies biomarkers in retinas, conducting investigations both *in vitro* and *in vivo*, using mouse models and human tissues. Muraleva and Kolosova (2023) investigated the molecular mechanism involved in AMD, focusing specifically on the contribution of alteration in the ERK1/2 signaling pathway. Their research suggests that in OXYS rats, the development of AMD-like pathology is accompanied by overactivation of the ERK1/2 signaling pathway. Additionally the progression of AMD-like pathology is correlated with the accumulation of pathological amyloid aggregates and tau hyperphosphorylation in the retina. Interestingly, the authors also found that in OXYS rats which spontaneously develop AMD-like and AD-like pathologies, ERK1/2 overactivation in the retina was paralleled by ERK1/2 hyperphosphorylation in the brain (Muraleva et al., 2019).

### 5.3 Oxidative stress

Oxidative stress has been suggested as a key factor for triggering RPE degeneration (Lin et al., 2011; Eells, 2019). It ultimately involves an excess of reactive oxygen species (ROS) mainly produced in mitochondria (Kaarniranta et al., 2020). Mitochondrial ROS and oxidative damage exhibit a notable increase with aging and age-related diseases. The initiation of the mitochondrial permeability transition pore, prompted by ROS and mitochondrial calcium overload, culminates in apoptosis (Rottenberg and Hoek, 2017). The increased ROS levels promote oxidative damage to mitochondrial DNA, lipids, and proteins (Jarrett et al., 2008). In the course of normal aging, disruptions in mitochondrial structure and accumulation of mutations in mitochondrial DNA have been documented (Blasiak et al., 2013b). These findings align with earlier studies by Wang et al. (2010), demonstrating an age-associated accumulation of mitochondrial DNA damage in the RPE and choroid of mice and rats. This effect was most likely caused by the aging-related reduction in DNA repair capacity, which was corroborated by the downregulation of genes encoding the enzymes MutY DNA glycosylase, thymine DNA glycosylase, and 8-oxoguanine-DNA glycosylase 1 (OGG1), which are essential for repairing oxidative damage to DNA. Moreover, PPARγ is reported to be expressed in RPE cells (Ershov et al., 2000; Choudhary et al., 2016). It is demonstrated that its agonists play an important role in preventing CNV and reducing dye leakage from CNVs in laser-induced CNV animal models (Datta et al., 2017), but also preserve greater cognitive function in AD patients than placebo (Watson et al., 2005). Moreover, an oxysterol, 27-hydroxycholesterol, appears to be implicated in oxidative damage both in AMD and AD. It would act by increasing the generation of reactive oxygen species (ROS) and the production of Aβ; additionally, the initiation of heme oxygenase 1 (HO-1), a protein that promotes the oxidation of cholesterol to oxysterols, is proposed as an early occurrence in the pathogenesis of sporadic AD. Notably, levels of HO-1 were also elevated in the RPE of maculas affected by AMD (Dasari et al., 2010). Furthermore, it is known that Aβ acts as a mitochondrial toxin, affecting the neurosynaptic pool by inducing oxidative stress in RPE cells (Butterfield and Boyd-Kimball, 2004; Ambroggio et al., 2005; Bruban et al., 2009; Wu et al., 2017). In the Bruban et al.’s (2009) study, the mechanisms of damage caused by the oligomeric form of Aβ (1–42) – O Aβ (1–42) – were investigated. O Aβ (1–42) reduced the mitochondrial redox potential and increased the production of reactive oxygen species. Additionally, it disorganized the actin cytoskeleton and decreased occludin expression by half, significantly reducing attachment capacity and eliminating the selectivity of RPE cell transepithelial permeability. However, the author also demonstrated that the use of antioxidant substances can partially reverse the effect of these two isoforms of beta-amyloid peptide.

This observation underscores an interconnection between the cited mechanisms in the physiopathology of AMD and Tauopathies, indicating a mutual association. Subsequent sections will provide a detailed examination of these shared mechanisms.

## 6 Senescence as intermediate key to tauopathies

According to the findings shown above, oxidative stress is a major factor in AMD pathogenesis, inducing a range of DNA lesions, with 8-oxo-7,8-dihydroguanine (8-oxoG) functioning as a marker of oxidative DNA damage and a prominent mutagenic mediator of oxidative stress (Kushwah et al., 2023). Previous studies discovered that lymphocytes from AMD patients have more endogenous DNA damage than lymphocytes from healthy persons (Blasiak et al., 2013a).

The RPE cells can undergo oxidative stress-induced senescence despite remaining dormant. They proliferate (Glotin et al., 2008; Marazita et al., 2016). In fact, several studies that used human RPE-derived ARPE-19 cells that proliferate *in vitro* showed how oxidative stress causes cell senescence. Cellular senescence is caused by persistent DNA damage response (DDR) orchestrated by the serine/threonine-protein kinases ATM (ataxia telangiectasia mutated) and ATR (ataxia telangiectasia and Rad3-related), which are the major cell sensors of DNA damage (Pezone et al., 2023). On the other hand, oxidative damage restricts the replication and transcription of mitochondrial DNA (mtDNA), diminishing mitochondrial function and leading to a rise in ROS production, further mtDNA damage, and senescence (Correia-Melo and Passos, 2015; Perillo et al., 2020).

DNA damage response is a complicated signal-transduction process that detects DNA lesions (SSBs and DSBs) and organizes brief cell-cycle arrest, DNA repair, autophagy, apoptosis, or permanent growth arrest (senescence) depending on their severity (De Zio et al., 2013; Feringa et al., 2018; Lanz et al., 2019; Kargapolova et al., 2021). It depends on activating of sensor kinases (ATM, ATR, and DNA-dependent protein kinase). If DNA damage continues, the senescence phenotype emerges gradually through the induction of checkpoint proteins such as p53, p21, and p16, which induce cell-cycle arrest and senescence (Medzhitov, 2021). Surprisingly, this anti-proliferative response (induction of p16 and p21) is also present in non-replicating postmitotic cells and stem cells following DNA damage to maintain cell survival (Yosef et al., 2017; Kumari and Jat, 2021; Von Zglinicki et al., 2021).

Senescence is induced by a persistent DNA damage response that reduces the NAD+ pool and favors the senescence-associated secretory phenotype (SASP) and mitochondrial malfunction. Although some inconsistent interpretations imply that SASP has either favorable or negative effects on the course of age-related illnesses or neoplasia, the relationship between SASP and DDR is well established (Gorgoulis et al., 2019; Pezone et al., 2023). DDR-induced inflammatory phenotypes, which have defense and messaging roles in complex cell populations, might, on the other hand, result in a prolonged inflammatory milieu that promotes the accumulation of DNA mutations and genomic instability. Persistent inflammation and DDR are the molecular processes that underpin genomic and chromosomal instability, culminating in the reactivation of repressed genes in aged neurons (Morano et al., 2014; Russo et al., 2016, 2021; Tijhuis et al., 2019). DNA methylation and PRC complex binding loss or increase are found in aged cells. Mechanistically, homology-directed DSB repair leads to strand-specific editing of local methylation (Allen et al., 2017; Tijhuis et al., 2019; Tramontano et al., 2020) resulting in cells with hypermethylated and hypomethylated DNA (Sandovici et al., 2006; O’Hagan et al., 2008) and global transcriptional reprogramming (Pezone et al., 2017; Xiao et al., 2019; Improda et al., 2023).

DNA damage response processes work to repair DNA damage. However, if a cell’s DNA is seriously damaged, the cell will either go into dormancy or will undergo programmed cell death. Autophagy affects a cell’s destiny following DNA damage by acting as a prosurvival process as well as a kind of cell death. According to some research, autophagy delays DNA damage-induced apoptosis by supplying energy for DNA repair (Demirbağ-Sarikaya et al., 2021). By removing harmful aggregates that might be a source of ROS, autophagy often supports DDR and indirectly reduces DNA damage (Blasiak et al., 2017).

Numerous DDR proteins have a role in controlling autophagy. To repair DNA single-strand breaks, PARP1 catalyzes the polyribosylation of nuclear proteins by converting NAD+ into polyADP-ribose polymers. As a result, the energetic imbalance that triggers autophagy through the AMPK pathway, recycling metabolic precursors for ATP and providing energy for DDR. This results in NAD+ depletion and ATP use (Muñoz-Gámez et al., 2009). As model organisms age, autophagy often decreases, leading to a buildup of cellular waste and browning of the cells (Rubinsztein et al., 2011).

Retinal pigmented epithelium cell senescence may also be directly associated with mitochondrial malfunction. Dysfunctional mitochondria lead to higher amounts of ROS, mtDNA damage, and impaired metabolic performance. The transcriptional coactivator peroxisome proliferator-activated receptor-gamma (PGC-1α) is essential for mitochondrial biogenesis and oxidative metabolism. Recently DNA sequence variants in PPARGC1A gene coding for PGC-1α were reported to be associated with neovascular AMD and AMD-associated loci (Gurubaran et al., 2023). Moreover, PGC-1α has been found to influence lysosomal activity in neurons and RPE cells, increasing autophagy flux and eliminating cell damage (Tsunemi et al., 2012). In same study, it was also shown that PGC-1 defective animals exhibited several RPE abnormalities, which were connected with their accelerated senescence (Kaarniranta et al., 2018).

In summary, irreversible pathogenic processes including RPE loss and inflammation may be aided by or preceded by RPE senescence in the retina, which is peculiar to AMD. Senescent RPE cells may become excessively harmed and dysfunctional due to SASP overexpression.

## 7 Future directions

The neural retina, being a component of the brain, endures the same changes as the aging brain. The brain is prone to aging, which appears as changes in its structure and cognitive processes (Wrigglesworth et al., 2022). In AMD and AD pathogeneses share several risk factors such as age, obesity, atherosclerosis, and hypertension (Kaarniranta et al., 2011) but share also several mechanisms, such as inflammation, tau hyperphosphorylation, oxidative stress, drusen and alternative complement cascade activation (Meri and Haapasalo, 2020). Aβ plaques, a hallmark of AD, can then be present in AMD drusen and may enhance complement activation by blocking complement factor I (CFI) (Mirzaei et al., 2020).

Alzheimer’s disease brains are distinguished by neuronal death and the formation of plaques containing A fibrils. Nonetheless, several studies imply that soluble Aβ species may be implicated in pathogenic AD episodes (Miller-Thomas et al., 2016). Drusen, extracellular deposits formed by the aged retina, are similar to senile plaques found in the aging brain.

In cell cultures, experimental animals, and human post-mortem eyes, experimental exposure of the neural retina, RPE, and choroid to Aβ resulted in local retinal inflammation. In rat retinas (Lynn et al., 2017; Pezone et al., 2023), Aβ was shown to accumulate in lysosomes, affecting RPE function and producing an AMD-like phenotype (Lynn et al., 2017). These effects might be linked to lysosomal clearance impairment caused by aging and AMD (Kaarniranta et al., 2018).

Beta-amyloid has been shown to promote RPE cell senescence as well as to disrupt mitochondrial metabolism (Muraleva et al., 2019). Both effects have been identified as involved in the etiology of AMD (Blasiak, 2020).

Senescent cells’ production of SASP-derived substances may cause low-grade inflammation, which is critical in developing AMD and other neurological disorders (Carreno et al., 2021).

Senescent alterations in the expression pattern of several proteins may result in microglia activation and structural abnormalities associated with AMD (Indaram et al., 2015).

**FIGURE 2:**
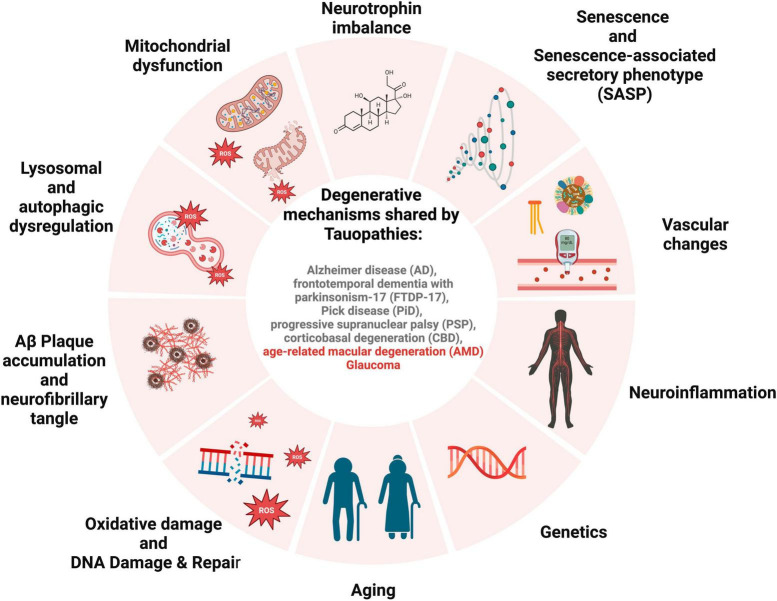
Schematic for overlapping degenerative mechanisms shared by tauopathies. These pathways have been associated with brain and retinal degeneration, cognitive impairment, and loss of vision.

Al-Hussaini et al. (2008) suggested that RPE cells in the central retina stay quiescent due to space restrictions and contact with neuroretina, and when wounded, they can be replaced by their developing counterparts at the RPE periphery in an endogenous compensatory mechanism. This endogenous regeneration mechanism is increased in pathogenic circumstances, which may deteriorate with age (Xia et al., 2011). If RPE cells are prone to senescence, it can lead to the inability of peripheral RPE cells to rescue their central RPE counterparts, resulting in a significant loss of RPE cells in clinically evident AMD. This pathway may malfunction and lead to AMD if the majority of the macular peripheral RPE cells are impacted by senescence. Senescent RPE will be the disease’s cause, negatively impacting surrounding tissue through SASP (Figure 2).

In conclusion, oxidative damage, Aβ deposits and phosphorylated tau may play an important role in the pathogenesis of AMD and serve as a reliable measure of real retina aging with potential applications in diagnosis and treatment. These factors may be connected to oxidative stress, decreased mitochondrial and lysosomal function, inflammation, and particular complement system genotypes, among other consistent aspects of AMD development. Aβ deposits may be seen in both dry and wet AMD, and several pathways may highlight the negative consequences of Aβ deposition in AMD pathogenesis (Kaarniranta et al., 2020). Systemic and ocular levels of oxidative damage are currently evaluated as diagnostic and/or even predictive biomarkers of both AMD and tauopathies (Blasiak et al., 2013b).

Most current treatments temporarily prevent tissue degradation by reducing inflammation or complement dysregulation using anti-inflammatory, antioxidant, or antiangiogenic medications. However, based on similarities between AMD and tauopathies, an increasing number of opportunities are emerging with the administration of specific senolytics targeting aging cells (Chung and Kim, 2022) as a common therapeutic agent and neuroprotective strategy for AMD and tauopathies. Currently, a pilot clinical trial has been made to evaluate the effectiveness of senolytic therapy in modulating the progression of AD, which drives cellular senescence in the brain (Chung and Kim, 2022).

We believe that this observation will aid many other degenerative eye disorders (such as ocular scarring pemphigoid, OCP, or glaucoma) (Passaro et al., 2023) by elucidating the shared pathophysiology (DNA damage and inflammation associated with senescence).

## 8 Methods of search

For this review, an extensive exploration of literature was conducted from October 2023 to February 2024, utilizing the PubMed, Scopus, and Cochrane databases. No time limits were set in the searches. Our search methodology comprised a blend of keywords aimed at encompassing pertinent studies, such as “neurodegeneration,” “retina,” “maculopathy,” “AMD,” “tau,” “tauopathy,” and combinations of them. The articles identified underwent meticulous review and analysis, synthesizing a thorough understanding of what is known in this domain of study. Figures are made in BioRender.com.

## Author contributions

MR: Conceptualization, Writing – review & editing. APe: Conceptualization, Supervision, Writing – review & editing. GQ: Investigation, Writing – review & editing. GA: Writing – review & editing. ML: Writing – review & editing. MP: Writing – review & editing. APo: Supervision, Writing – review & editing. CC: Project administration, Supervision, Writing – review & editing.
